# Plasma proteome and metabolome characterization of an experimental human thyrotoxicosis model

**DOI:** 10.1186/s12916-016-0770-8

**Published:** 2017-01-09

**Authors:** Maik Pietzner, Beatrice Engelmann, Tim Kacprowski, Janine Golchert, Anna-Luise Dirk, Elke Hammer, K. Alexander Iwen, Matthias Nauck, Henri Wallaschofski, Dagmar Führer, Thomas F. Münte, Nele Friedrich, Uwe Völker, Georg Homuth, Georg Brabant

**Affiliations:** 1 Institute of Clinical Chemistry and Laboratory Medicine, University Medicine Greifswald, Ferdinand-Sauerbruch-Straße, 17475 Greifswald, Germany; 2 DZHK (German Centre for Cardiovascular Research), partner site Greifswald, Greifswald, Germany; 3 Department of Functional Genomics, Interfaculty Institute for Genetics and Functional Genomics, University Medicine and Ernst-Moritz-Arndt University Greifswald, Friedrich-Ludwig-Jahn-Straße 15a, D-17475 Greifswald, Germany; 4 Medical Clinic I, University of Lübeck, Experimental and Clinical Endocrinology, Ratzeburger Allee 160, Zentralklinikum (Haus 40), 23538 Lübeck, Germany; 5 Private Practice Endocrinology, Krämpferstraße 6, 99094 Erfurt, Germany; 6 Department of Endocrinology and Metabolism, University Hospital Essen, University Duisburg-Essen, Hufelandstraße 55, 45122 Essen, Germany; 7 Department of Neurology, University of Lübeck, Ratzeburger Allee 169, 23538 Lübeck, Germany; 8 Research Centre for Prevention and Health, Glostrup University Hospital, Nordre Ringvej 57, 2600 Glostrup, Denmark; 9 ZIK-FunGene (Zentrum für Innovationskompetenz - Funktionelle Genomforschung), Greifswald, Germany

**Keywords:** Hyperthyroidism, Metabolomics, Proteomics, Thyroid function, Thyrotoxicosis

## Abstract

**Background:**

Determinations of thyrotropin (TSH) and free thyroxine (FT_4_) represent the gold standard in evaluation of thyroid function. To screen for novel peripheral biomarkers of thyroid function and to characterize FT_4_-associated physiological signatures in human plasma we used an untargeted OMICS approach in a thyrotoxicosis model.

**Methods:**

A sample of 16 healthy young men were treated with levothyroxine for 8 weeks and plasma was sampled before the intake was started as well as at two points during treatment and after its completion, respectively. Mass spectrometry-derived metabolite and protein levels were related to FT_4_ serum concentrations using mixed-effect linear regression models in a robust setting. To compile a molecular signature discriminating between thyrotoxicosis and euthyroidism, a random forest was trained and validated in a two-stage cross-validation procedure.

**Results:**

Despite the absence of obvious clinical symptoms, mass spectrometry analyses detected 65 metabolites and 63 proteins exhibiting significant associations with serum FT_4_. A subset of 15 molecules allowed a robust and good prediction of thyroid hormone function (AUC = 0.86) without prior information on TSH or FT_4_. Main FT_4_-associated signatures indicated increased resting energy expenditure, augmented defense against systemic oxidative stress, decreased lipoprotein particle levels, and increased levels of complement system proteins and coagulation factors. Further association findings question the reliability of kidney function assessment under hyperthyroid conditions and suggest a link between hyperthyroidism and cardiovascular diseases via increased dimethylarginine levels.

**Conclusion:**

Our results emphasize the power of untargeted OMICs approaches to detect novel pathways of thyroid hormone action. Furthermore, beyond TSH and FT_4_, we demonstrated the potential of such analyses to identify new molecular signatures for diagnosis and treatment of thyroid disorders. This study was registered at the German Clinical Trials Register (DRKS) [DRKS00011275] on the 16th of November 2016.

**Electronic supplementary material:**

The online version of this article (doi:10.1186/s12916-016-0770-8) contains supplementary material, which is available to authorized users.

## Background

Thyroid hormones (TH) circulating as thyroxine (T_4_) and triiodothyronine (T_3_) are essential for normal development and function of virtually all tissues [1]. Both their synthesis and release are closely controlled by pituitary thyroid stimulating hormone (TSH), which in turn is stimulated by hypothalamic thyrotropin releasing hormone (TRH). TH exert a negative feedback on synthesis and secretion of TRH as well as of TSH. As the feedback of TH on the hypothalamic-pituitary regulation of TSH is particularly sensitive, the robust relation of TSH and free T_4_ (FT_4_) is generally used as the ‘gold standard’ tool for diagnosis and follow-up of thyroid disorders.

Specific TH transporters mediate the cellular uptake of TH [2]. At the latest in the target cells, specific deiodinases convert T_4_ to T_3_ which is the major ligand for the nuclear TH receptors (TR) α and β and their subtypes [1]. Formation of ligand-activated TR homodimers and heterodimers with TR auxiliary proteins and other receptors, such as retinoid X receptor (RXR), finally results in stimulated or repressed expression of TH target genes. In addition to this so-called genomic action mediated by nuclear TRs, TH exert rapid non-genomic effects by binding to extranuclear receptors, like truncated cytoplasmic TRα isoforms or plasma membrane-localized integrin ανβ3, resulting in the activation of specific phosphorylation cascades [3]. Also, for the putative TH derivative 3,5-diiodothyronine, interaction with specific mitochondrial sites was reported [3]. Thus, in sum, by cell- and organ-specific TH uptake and activation, TR subtype synthesis and non-genomic modulation, TH are able to induce their various tissue- and cell-specific responses. It is thus not surprising that clinical symptoms of thyroid dysfunction are regarded to be of restricted diagnostic value because they are neither sufficiently sensitive nor specific [4]. Currently, the diagnosis of thyroid dysfunction and the assessment of treatment effects are almost entirely based on the biochemical determination of serum TSH, free T_4_ (FT_4_) and, under special conditions, free T_3_ (FT_3_). However, their use is limited by a number of drawbacks.

Despite the sensitive negative feedback regulation between TSH and FT_4_ leading to a tightly controlled individual set point [5, 6], large population-based studies established a wide reference range for TSH and free TH levels. This is explained by varying sensitivity at different levels of the activation process as well as the negative feedback mechanisms [7] and differences between assay specificities [8, 9]. Additionally, a number of rare severe clinical conditions lead to discordant alterations in serum TSH and FT_4_, including resistance to TH, TSH producing pituitary tumors, or central hypothyroidism [10, 11]. Therefore, peripheral biomarkers such as cholesterol and sex hormone- binding globulin (SHBG) concentrations have been suggested under these conditions as they are strongly correlated with thyroid function [12, 13]. However, because these parameters are also influenced by non-thyroidal disturbances, they were never established in clinical practice and accordingly current guidelines do not recommend their use [14]. Thus, currently available diagnostic tools are insufficient and novel biomarkers are urgently needed.

Indeed, systematic screens for novel markers of thyroid function in humans are lacking so far. In particular, only few studies attempted to detect peripheral TH effects by untargeted approaches. The influence of thyroid dysfunction on various tissue-specific proteomes or the metabolomes of serum and urine was assessed almost entirely using rodent models [15–18]. Even if these studies undoubtedly added to our understanding of TH action on metabolism, translation of these results to humans is still missing. Moreover, most of the scarce data on peripheral TH effects in humans are based on observations in patients with thyroid disorders such as autoimmune thyroid disease, which hamper the distinction between TH dependent effects and those related to the underlying autoimmune process. To avoid these problems, we herein studied TH effects in a strictly controlled model of experimental hyperthyroidism where healthy young male volunteers were subjected to a challenge of thyroxine over a period of 8 weeks. Untargeted plasma proteome and metabolome analyses were performed in a hypothesis-free approach to detect FT_4_-associated proteins and metabolites, and the generated data were used for characterization of main physiological signatures and to develop a biomarker-based classification model that allows prediction of TH function without prior information on TSH or free TH.

## Methods

### Study design and sampling

**Fig. 1:**
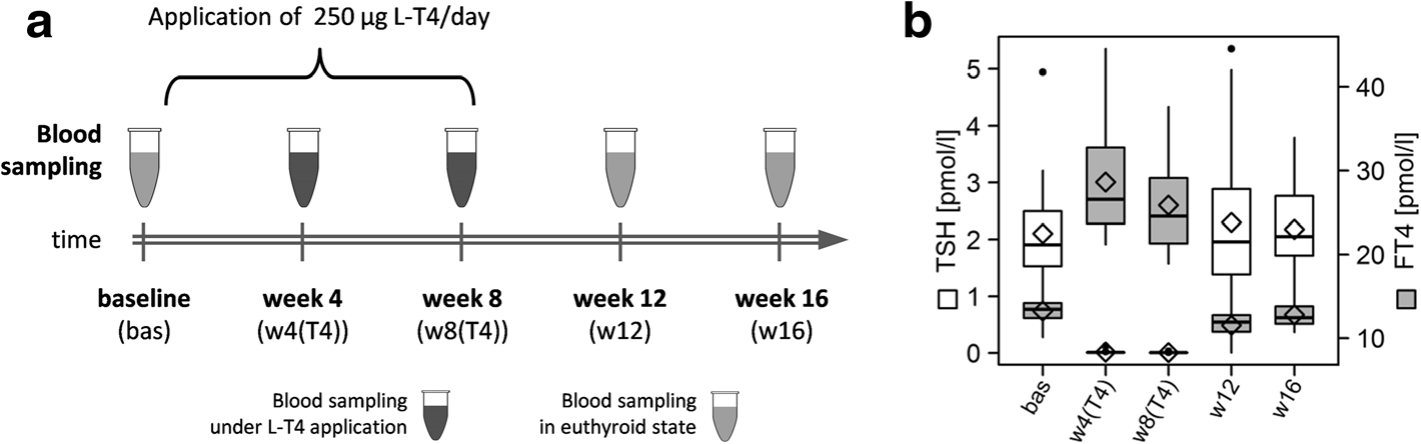
**a** Study design including sampling time points as well as duration of levothyroxine (L-T_4_) treatment. **b** Boxplots with mean values (﻿diamonds) of serum TSH (white) and FT_4_ (grey) for each time point. *bas* baseline, *w4(T4)/w8(T4)* 4 and 8 weeks of levothyroxine treatment, *w12/w16* 4 and 8 weeks after stopping the application point

Sixteen young healthy male subjects were treated with a single tablet of 250 μg levothyroxine (L-T_4;_ Henning-Berlin, Berlin, Germany) per day for 8 weeks. Plasma was sampled before L-T_4_ intake started (baseline, bas), after 4 (w4(T4)) and 8 (w8(T4)) weeks under treatment as well as 4 (w12) and 8 (w16) weeks after ending the application, respectively (Fig. 1a). The chosen sample size is appropriate as the volunteers were selected to reduce inter-individual variance. The repeated measure character of the study further reduced the influence of inter-individual variance. Body mass index (BMI) of the volunteers ranged from 21 to 30 kg/m^2^ and their age from 22 to 34 years (Table 1). During the study, thyrotoxicosis questionnaires were performed as well as 24 h blood pressure, and pulse rate activity (Cambridge Nanotechnology, Cambridge, UK) were recorded. The work has been approved by the ethics committee of the University of Lübeck and written informed consent was received from all participants prior to the study. The study conformed to the WMA Declaration of Helsinki.

**Table 1:** Clinical characteristics of participants during the study period

Characteristics	baseline	4 weeks (L-T4)^a^	8 weeks (L-T4)^a^	12 weeks	16 weeks	β (SD)^b^	Log^c^	*P* value(SD)^d^
Age, years	27.8 (3.8)	27.8 (3.8)	27.8 (3.8)	27.8 (3.8)	27.8 (3.8)	–	–	–
BMI, kg/m^2^	24.1 (2.4)	24.1 (2.4)	24.1 (2.4)	24.1 (2.4)	24.1 (2.4)	–	–	–
FT_4_, pmol/L	13.2 (1.4)	28.6 (6.5)	25.9 (5.7)	11.5 (1.5)	12.8 (1.5)	–	–	<0.001^e^
FT_3_, pmol/L	5.27 (0.51)	9.19 (2.01)	8.92 (2.25)	4.61 (0.33)	4.86 (0.55)	2.76 × 10^–1^ (8.22 × 10^–3^)	no	7.43 × 10^–25^ (4.28 × 10^–24^)^f^
TSH, mU/L	2.104 (1.017)	0.017 (0.029)	0.007 (0.007)	2.298 (1.309)	2.177 (0.897)	–1.35 × 10^–1^ (6.42 × 10^–3^)	yes	7.26 × 10^–21^ (2.02 × 10^–20^)^f^
SHBG, nmol/L	30.2 (10.2)	50.6 (16.2)	55.9 (16.3)	36.3 (11.8)	29.3 (9.3)	1.41 × 10^–2^ (9.11 × 10^–4^)	yes	3.41 × 10^–10^ (1.03 × 10^–9^)^f^
Cystatin C, mg/L	0.68 (0.06)	0.79 (0.08)	0.86 (0.12)	0.71 (0.07)	0.68 (0.06)	4.19 × 10^–3^ (2.35 × 10^–4^)	yes	8.26 × 10^–9^ (1.10 × 10^–8^)^f^
Serum glucose, mmol/L	5.18 (0.35)	5.22 (0.42)	5.26 (0.39)	5.09 (0.31)	5.18 (0.57)	5.76 × 10^–3^ (1.62 × 10^–3^)	no	2.91 × 10^–1^ (1.38 × 10^–1^)
Insulin, μU/L	8.35 (3.63)	7.94 (4.32)	7.78 (3.62)	8.33 (4.06)	8.07 (3.38)	–1.29 × 10^–3^ (1.14 × 10^–3^)	yes	6.58 × 10^–1^ (2.29 × 10^–1^)
HDL-cholesterol, mmol/L	1.43 (0.27)	1.21 (0.20)	1.23 (0.25)	1.46 (0.29)	1.42 (0.37)	–1.30 × 10^–2^ (8.89 × 10^–4^)	no	1.33 × 10^–5^ (1.54 × 10^–5^)^f^
LDL-cholesterol, mmol/L	2.70 (0.72)	2.15 (0.57)	2.27 (0.53)	2.91 (0.75)	2.76 (0.79)	–4.18 × 10^–2^ (1.96 × 10^–3^)	no	2.91 × 10^–8^ (6.08 × 10^–8^)^f^
Cholesterol, mmol/L	4.53 (0.75)	3.81 (0.61)	4.06 (0.61)	5.04 (0.70)	4.61 (0.68)	–5.87 × 10^–2^ (2.24 × 10^–3^)	no	3.05 × 10^–10^ (6.84 × 10^–10^)^f^
Triglycerides, mmol/L	1.26 (0.76)	1.14 (0.58)	1.29 (0.56)	1.31 (0.63)	1.35 (0.82)	–1.96 × 10^–3^ (6.06 × 10^–4^)	yes	3.08 × 10^–1^ (1.65 × 10^–1^)
ALT, μkatal/L	0.51 (0.21)	0.38 (0.10)	0.65 (0.42)	0.61 (0.29)	0.50 (0.14)	–4.30 × 10^–3^ (9.65 × 10^–4^)	yes	9.99 × 10^–2^ (7.00 × 10^–2^)
AST, μkatal/L	0.49 (0.41)	0.34 (0.14)	0.43 (0.17)	0.43 (0.14)	0.43 (0.23)	–2.03 × 10^–3^ (1.30 × 10^–3^)	yes	4.67 × 10^–1^ (2.37 × 10^–1^)
GGT, μkatal/L	0.41 (0.09)	0.45 (0.11)	0.49 (0.11)	0.45 (0.15)	0.43 (0.11)	6.84 × 10^–4^ (8.20 × 10^–4^)	yes	6.55 × 10^–1^ (3.18 × 10^–1^)
Total bilirubin, μmol/L	12.5 (8.4)	12.6 (8.7)	13.5 (7.3)	11.9 (6.4)	11.9 (7.3)	1.56 × 10^–3^ (1.18 × 10^–3^)	yes	5.83 × 10^–1^ (2.35 × 10^–1^)
Direct bilirubin, μmol/L	2.86 (1.35)	3.04 (1.35)	3.28 (1.24)	2.84 (1.03)	3.03 (1.39)	3.00 × 10^–3^ (5.14 × 10^–4^)	yes	1.06 × 10^–1^ (6.69 × 10^–2^)
Complement C3, g/L	1.15 (0.27)	1.21 (0.16)	1.17 (0.11)	1.10 (0.14)	1.11 (0.14)	4.49 × 10^–3^ (5.06 × 10^–4^)	no	2.38 × 10^–2^ (1.75 × 10^–2^)^f^
Complement C4, g/L	0.24 (0.06)	0.25 (0.05)	0.24 (0.05)	0.23 (0.05)	0.24 (0.05)	9.81 × 10^–4^ (1.38 × 10^–4^)	no	3.09 × 10^–2^ (2.94 × 10^–2^)^f^

^a^ *L-T4* application of levothyroxine

^b^Mean and standard deviation (SD) of the estimate for FT_4_ in linear mixed regression models adjusted for age and body mass index (BMI) from 101 subsamples

^c^Dependent variable was logarithmized to base 10

^d^Mean and SD of the *P* value

^e^Repeated measurement analysis of variance adjusted for age and BMI

^f^Significant results

*FT*_*4*_ free thyroxine, *FT*_*3*_ free triiodothyronine, *TSH* thyrotropin, *SHBG* sex hormone binding globulin, *HDL* high-density lipoprotein, *LDL* low-density lipoprotein, *ALT* alanine aminotransferase, *AST* aspartate aminotransferase, *GGT* γ-glutamyl transpeptidase

### Assays

Serum levels of TSH, free triiodothyronine (FT_3_) and FT_4_ were measured using an immunoassay (Dimension VISTA, Siemens Healthcare Diagnostics, Eschborn, Germany) with a functional sensitivity of 0.005 mU/L for TSH, 0.77 pmol/L for FT_3_, and 1.3 pmol/L for FT_4_. SHBG levels were determined via a chemiluminescent enzyme immunoassay on an Immulite 2000XPi analyzer (SHBG Immulite 2000, Siemens Healthcare Medical Diagnostics, Bad Nauheim, Germany) with a functional sensitivity of 0.02 nmol/L. Serum cystatin C (CYTC) was measured using a nephelometric assay (Dimension VISTA, Siemens Healthcare Diagnostics, Eschborn, Germany) with a functional sensitivity of 0.05 mg/L. Insulin serum concentrations were measured using a chemiluminescent immunometric assay (Immulite 200 XPi; Siemens Healthcare Diagnostics) with a functional sensitivity of 2 mU/L. Lipids (total cholesterol, HDL- and LDL cholesterol, triglycerides), serum glucose, serum activities of alanine amino transferase (ALT), aspartate amino transferase (AST), γ-glutamyl transpeptidase (GGT), as well as the levels of the complement factors C3 and C4 were measured by standard methods (Dimension VISTA, Siemens Healthcare Diagnostics, Eschborn, Germany).

### Plasma metabolome analysis

Metabolic profiling of plasma samples was performed by Metabolon Inc. (Durham, NC, USA), a commercial supplier of metabolic analyses. Three separate analytical methods (GC-MS and LC-MS (positive and negative mode)) were used to detect a broad metabolite panel [19]. Briefly, proteins were precipitated from 100 μL plasma with methanol, which further contained four standards to monitor extraction efficiency, using an automated liquid handler (Hamilton ML STAR, Hamilton Company, Salt Lake City, UT, USA). The resulting extract was divided into four aliquots; two for analysis by LC, one for analysis by GC, and one reserve aliquot. Aliquots were placed briefly on a TurboVap® (Zymark, Sparta, NJ, USA) to remove the organic solvent. Each aliquot was then frozen and dried under vacuum. LC-MS analysis was performed on a LTQ mass spectrometer (Thermo Fisher Scientific Inc., Waltham, MA, USA) equipped with a Waters Acquity UPLC system (Waters Corporation, Milford, MA, USA). Two aliquots were reconstituted either with 0.1% formic acid (positive mode) or 6.5 mM ammonium bicarbonate (negative mode). Two separate columns (2.1 × 100 mm Waters BEH C18 1.7 μm particle) were used for acidic (solvent A: 0.1% formic acid in H_2_O, solvent B: 0.1% formic acid in methanol) and basic (A: 6.5 nM ammonium bicarbonate pH 8.0, B: 6.5 nM ammonium bicarbonate in 98% methanol) mobile phase conditions, optimized for positive and negative electrospray ionization, respectively. After injection, the samples were separated in a gradient from 100% A to 98% B. The MS analysis alternated between MS and data-dependent MS/MS scans using dynamic exclusion. GC-MS analysis was performed on a Finnigan Trace DSQ fast-scanning single-quadrupole mass spectrometer (Thermo Fisher Scientific Inc., Waltham, MA, USA), equipped with a GC column containing 5% phenyl residues. The temperature was ramped between 60 and 340 °C. For electron impact ionization one aliquot was derivatized under dried nitrogen using bistrimethyl-silyl-triflouroacetemide. Quality control of platform performance was achieved by the use of pooled samples and technical blanks as well as the addition of non-interfering internal standards to the samples. Metabolites were identified from LC-MS and GC-MS spectra by automated comparison with a proprietary library, containing retention times, m/z ratios, and related adduct/fragment spectra of over 1000 standard compounds measured by Metabolon. To correct for daily variations of platform performance, the raw area count of each metabolite was rescaled by the respective median value of the run day. In total, 380 metabolites could be identified.

### Plasma proteome analysis

Depletion of six highly abundant proteins in plasma was performed using multi-affinity chromatography (MARS6-human, Agilent Technologies, Waldbronn, Germany) in accordance with the manufacturer’s protocol. After precipitation of proteins of the non-bound fraction with trichloroacetic acid (final concentration 15%), the pellet was re-suspended in 100 μL 8 M urea/2 M thiourea. Protein concentrations of depleted samples were determined via a Bradford Assay (Bio-Rad Laboratories, Munich, Germany) using bovine serum albumin as standard protein. Individual protein samples (4 μg) were reduced with 2.5 mM dithiothreitol (60 °C, 1 h), subsequently alkylated with 10 mM iodoacetamide (37 °C, 30 min), and subjected to proteolytic cleavage with trypsin (Promega, Madison, WI, USA) using a trypsin to protein ratio of 1:25 overnight at 37 °C. After stopping the digestion with 1% acetic acid, samples were purified with C18 ZipTip® with a loading capacity of 2 μg (Millipore Cooperation, Billerica, MA, USA). Prior to MS analysis, desalted peptides were subjected to reverse phase chromatography. Chromatographic separation of peptides was done on a nanoAquity UPLC system equipped with a pre-column (nano Aquity UPLC Trap column, 180 μm × 20 mm, 5 μm) and reverse phase column (BEH130 C18, 100 μm × 100 mm, 1.7 μm) configuration (Waters Corporation, Milford, MA, USA). A 100-min non-linear gradient of 2–60% ACN in 0.1% acetic acid was run at a constant flow rate of 0.4 μL/min. Mass spectral data were recorded on-line on a LTQ-Orbitrap Velos mass spectrometer (Thermo Electron, Bremen, Germany) which was operated in a data-dependent acquisition mode. MS/MS fragmentation was performed by collision induced dissociation. The recorded LC-MS/MS raw data were processed using the Refiner MS software version 7.6.6 (GeneData, Basel, Switzerland) with an adapted workflow with the following steps: (1) chemical noise removal, (2) retention time alignment across all samples, and (3) feature extraction and isotope group clustering. Data was searched against a human Swissprot/Uniprot database (rel. 2012/08) limited to human entries with a precursor ion tolerance set to 10 ppm (0.6 Da for fragment ions) using an in-house MASCOT server (rel. 2.3). The carbamidomethylation of cysteine was set as static modification, methionine oxidation was considered as dynamic modification. Peptides identified with rank = 1 and an ion score ≥ 20 and identified as unique in the data set were used for relative quantitation on the level of summed peptide intensities per protein. MS analyses of all 80 plasma samples revealed 2374 unique peptides representing 497 human proteins. The mass spectrometry proteomics data have been deposited to the ProteomeXchange Consortium via the PRIDE [20] partner repository with the dataset identifier PXD004815 and 10.6019/PXD004815.

### Statistical analysis

To ensure a median availability of data points on three time points, only metabolites and proteins with less than 40% missing values were used for the analysis, resulting in 349 metabolites and 437 proteins. Values of metabolite/protein intensities were log_10_-transformed. To account for compliance and intestinal resorption during L-T_4_ treatment we applied a mixed-effect linear regression model with serum FT_4_ concentrations as exposure and metabolite/protein concentrations as outcome. Since the study considered repeated measurements, serum FT_4_ was determined as a fixed effect whereas the study participant was the random effect in the model. All analyses were adjusted for baseline age and BMI as well as experimental batch in case of proteome analyses. To account for multiple testing, we adjusted the *P* values of the regression analysis by controlling the false discovery rate (FDR) at 5% [21]. Distributional assumptions were tested visually using QQ-plots and no obvious violations were observed. Robustness of the results was assessed by a leave-three-out procedure. For this purpose, we randomly chose three participants and excluded them from the analyses. This procedure was repeated 100 times. Since three participants strikingly differed in their response to L-T_4_ regarding their serum ALT and AST activities, an additional data set was created excluding them leading to finally 101 distinct subsets of the data. Subsequently, estimates and FDR values were averaged across the subsamples. Metabolites and proteins with an average FDR below 0.05 were defined significant. In consequence, the results presented in this work constitute the most robust FT_4_-associated alterations. The functional classification analysis for significantly altered proteins was performed using Ingenuity Pathway Analysis software (Ingenuity Systems, Redwood City, CA, USA). Significance of the enrichment of altered proteins among functional categories was assessed by Fisher’s exact test. For every time point, ratios to the baseline values for each participant were calculated and plotted as mean log_2_-fold change.

### Sample classification

For classification purposes, samples were divided in two groups. First, all samples before L-T_4_ treatment as well as 8 weeks after cessation of treatment were defined as euthyroid. Second, all samples from the two treatment time points were defined as hyperthyroid. Both assignments were justified by suppressed serum TSH concentrations in concordance with elevated serum FT_4_ (Table 1 and Fig. 1). In total, 64 samples were used for classification analysis (time point w12, 4 weeks after stop of treatment, was left out because of the presence of an intermediate state). To ensure reproducibility of possible markers, only metabolites/proteins without missing values as well as unambiguous assignment were used, resulting in 201 metabolites and 207 proteins. Since no independent validation set was available and to avoid overfitting, we performed a two-stage cross validation procedure to select a subset of metabolites/proteins capable of classifying the samples using a random forest [22] as classifier (Additional file 1: Figure S1). A first split was performed to divide samples in training and validation set (outer loop; repeated 30 times). The resulting training sets were once more partitioned into training and test set (inner loop; repeated 50 times). Based on the last split, a random forest was trained. Prediction performance was assessed using receiver operating characteristic (ROC) curves on the independent test set for the current loop. Variable importance was assessed by the Gini index [23] for each feature of the trained forest. Variable importance of each inner loop were averaged weighted by the area under the ROC-curves (AUC). The 15 most important variables from the inner loop were taken forward to build a new random forest. Analogous to the previous procedure the prediction performance was assessed yielding the final classification performance based on a reduced subset of the features. The random forest was implemented in R via the randomForest package (v 4.6-10) [22]. Statistical analyses were performed using SAS version 9.4 (SAS statistical software, version 9.4, SAS Institute, Inc.; NC, USA) and R 3.0.1 (R Foundation for statistical computing, version 3.0.1, Vienna, Austria).

## Results and Discussion

### L-T_4_ treatment and standard clinical hormone assays

**Fig. 2:**
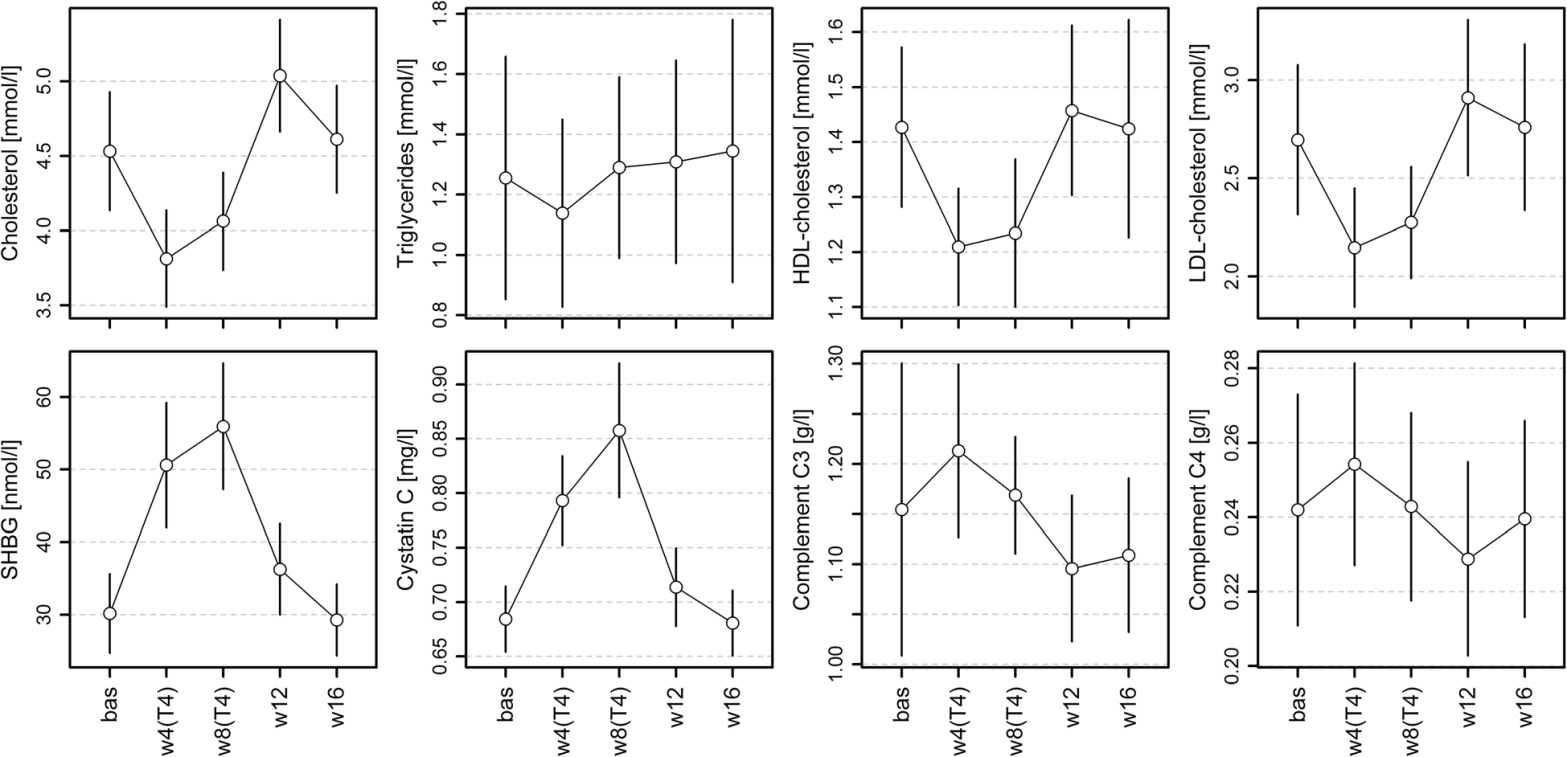
Means with 95% confidence intervals for serum concentrations of selected biochemical parameters during the study periods. Corresponding estimates from regression analyses are given in Table 1. *bas* baseline, *w4(T4)/w8(T4)* 4 and 8 weeks of levothyroxine treatment, *w12/w16* 4 and 8 weeks after stopping the application

As previously described [24], treatment with 250 μg/day L-T_4_ for 8 weeks resulted in the expected suppression of mean TSH concentrations from 2.10 mU/L (standard deviation (SD): ±1.01) at baseline to 0.017 mU/L (SD: ±0.029) at 4 weeks and 0.007 mU/L (SD: ±0.007) at 8 weeks, respectively. Mean concentrations of FT_4_ and FT_3_ exhibited the opposite profile with peak concentrations of 28.6 pmol/L (SD: 5.7) and 9.19 pmol/L (SD: ±2.01) after 4 weeks of L-T_4_ intake, respectively, consistent with a biochemical condition of overt hyperthyroidism (Fig. 1 and Table 1). All parameters normalized within the first 4 weeks after termination of L-T_4_ intake (Fig. 1b). We further assessed effects on some of the well-known TH targets such as SHBG, CYTC, and different blood lipids (Table 1). In general, L-T_4_ treatment resulted in a transient decline of blood lipids (Fig. 2) apart from triglycerides, whereas serum glucose and insulin were not significantly altered. The complement factors C3 and C4 showed a moderate, but significant positive association with FT_4_ (Fig. 2).

### General FT_4_-associated alterations of the plasma metabolome

**Fig. 3:**
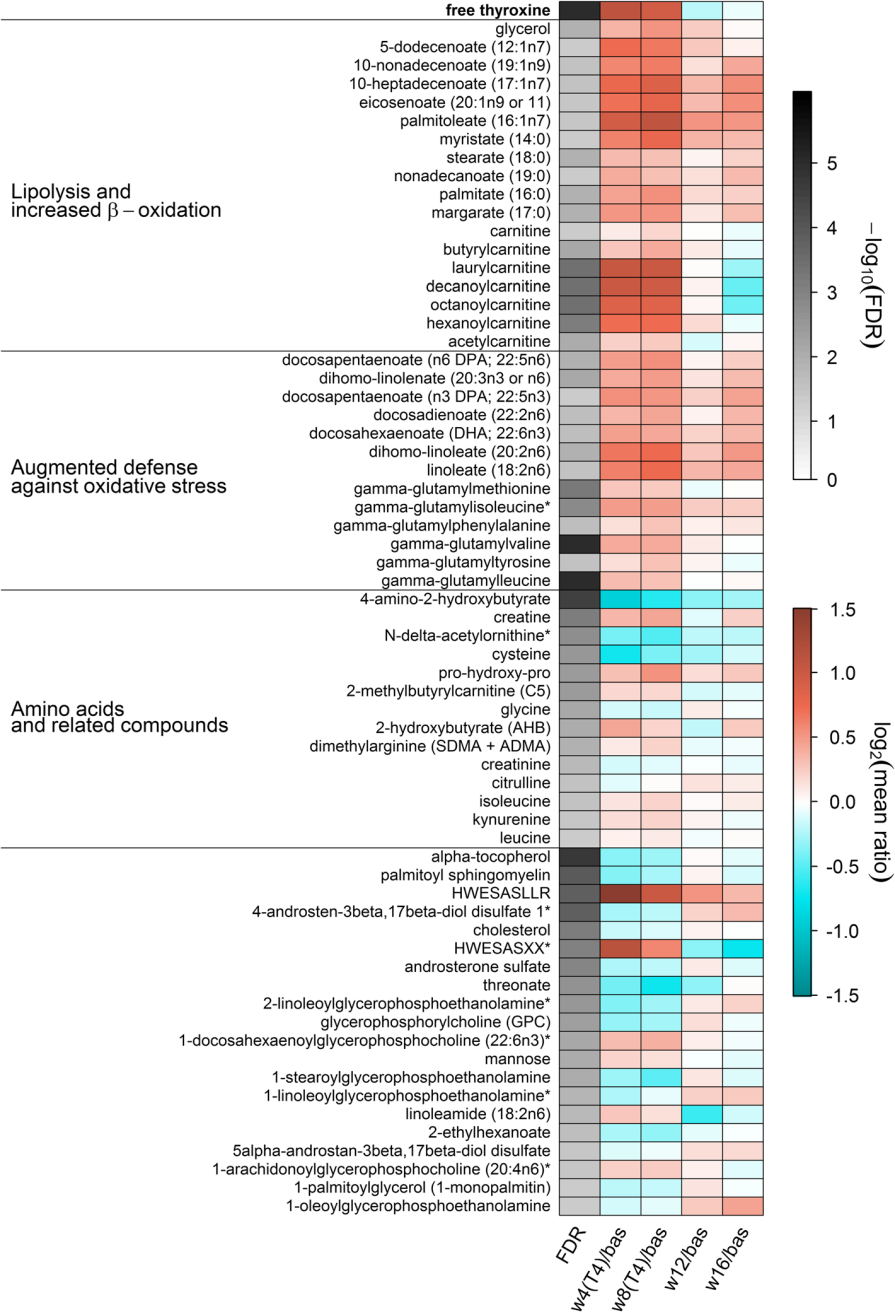
Heatmap of plasma metabolites significantly associated with free thyroxine (FT_4_) in mixed effect linear regression models. The first column displays the values of the mean false discovery rate (FDR) for the FT_4_ effect across 101 subsamples coded in grey color. The other columns indicate the mean log_2_-ratio from baseline (bas) compared to 4 (w4(T4)) and 8 (w8(T4)) weeks of treatment as well as 4 (w12) and 8 (w16) weeks after finishing the treatment. The time course of the FT_4_ concentrations is shown on top of the map as reference. Orange shading denotes an increase and blue shading a decrease compared to baseline, respectively. Derived physiological signatures are labeled on the left. The corresponding estimates and FDR values from regression analysis can be found in Additional file 2: Table S1. Metabolites marked with a star were assigned based on in silico fragmentation spectra

Treatment of the euthyroid male volunteers with L-T_4_ markedly affected the plasma metabolome, significantly altering the levels of 65 out of 349 detected metabolites (19%), of which 45 exhibited a positive and 20 a negative association with serum FT_4_, respectively. The associated metabolites represented diverse metabolite classes, where lipids and related compounds encompassed the largest portion of FT_4_-associated molecules (39 of 65 present in the analysis panel). These could be assigned to the following categories: free fatty acids (FFAs), acyl carnitines (ACs), polyunsaturated fatty acids (PUFAs), lysophospholipids (LPs), and androgens. All results are summarized in Fig. 3 and Additional file 2: Table S1.

### A plasma metabolome signature indicating increased resting energy expenditure and enhanced mitochondrial fatty acid β-oxidation

Thyroxine treatment induced an increase in long chain saturated as well as monounsaturated FFAs, which was accompanied by elevated glycerol levels (Fig. 3). As demonstrated by Mitchell et al. [25], both Graves’ thyrotoxicosis and resistance to TH due to *THRB* mutations (RTHβ) result in significantly increased resting energy expenditure. Involved mechanisms include TH-stimulated lipolysis in white adipose tissue, mediated by increased local concentrations of catecholamines with successive activation of adipocyte β-adrenergic receptors [26, 27]. Consistently, the ubiquitous increase of FFAs and glycerol in plasma following L-T_4_ treatment observed in the present study clearly indicates TH-triggered lipolysis in white adipose tissue.

After transport into tissues highly active in respiration, namely skeletal muscle as the major determinant of energy expenditure in humans [28], and liver, FFAs are subjected to mitochondrial β-oxidation that is also enhanced by TH. This is mainly mediated by increased expression of *CPT1* encoding carnitine palmitoyltransferase-I (CPT-1), which represents a direct transcriptional target of T_3_-activated TR [29]. In exchange with carnitine, acyl-carnitines are translocated through the outer mitochondrial membrane by CPT-1 [30]. Simultaneous TH-mediated up-regulation of the gene encoding the final enzyme of carnitine biosynthesis, the γ-butyrobetaine hydroxylase, ensures the increased carnitine levels required for that, as demonstrated in a rodent model [31]. The resulting enhanced activity of the complete carnitine acyl-carnitine translocase system explains the pronounced increase in short to medium chain ACs in plasma observed in this study (Fig. 3), as a fraction of the newly synthesized ACs spills from tissue in the circulation, as reported earlier by Jourdan et al. [32]. Indeed, our results replicate the positive association between ACs and FT_4_ reported by this group for the population-based KORA cohort study based on data from 1463 euthyroid individuals [32]. In contrast, a previous patient-based study revealed no relation between plasma AC profiles and the restoration of euthyroidism [33]. However, the value of the latter results might be limited by the small sample size of six hyper- and hypothyroid individuals, respectively.

Augmented β-oxidation of FFAs causes increased acetyl-CoA levels and stimulation of the TCA cycle, which would be expected to finally trigger increased ATP production by oxidative phosphorylation. Indeed, accelerated carbon flux through the TCA cycle was demonstrated in human skeletal muscle in a short-term model of experimental thyrotoxicosis (Lebon et al. [34]) as well as in patients suffering from RTHβ (Mitchell et al. [25]). However, ATP production by oxidative phosphorylation did not change under these conditions, which was explained by increased uncoupling of respiration and ATP synthesis, potentially caused by increased expression of UCP3 (SLC25A9) encoding the mitochondrial uncoupling protein 3 (Lebon et al. 2001; Mitchell et al. [25]). Less efficient ATP generation in muscle might also explain our observation of increased creatine plasma levels (Fig. 2) under conditions of thyrotoxicosis, as the synthesis of the central muscle energy storage compound phosphocreatine by ATP-dependent creatine phosphorylation can be predicted to be reduced by TH-induced mitochondrial uncoupling.

Enhanced systemic glucose utilization represents a further well-known consequence of increased energy expenditure under conditions of hyperthyroidism [29]. In the liver, TH stimulate glycogenolysis and gluconeogenesis mainly consuming gluconeogenic amino acids and glycerol while down-regulating glycolysis, resulting in a higher hepatic glucose output [35]. In this context, the already mentioned strongly increased plasma glycerol pool under L-T_4_ treatment that is explained by stimulated lipolysis also represents a potential source for gluconeogenesis (Fig. 3). Our did not support for the use of amino acids as a source for TH-stimulated hepatic gluconeogenesis, partially in line with results of the already mentioned population-based KORA study [32]. However, the unaltered plasma levels of gluconeogenic amino acids that were measured despite the predicted increased demand after L-T_4_-treatment could be caused by the enhanced renal amino acid recovery reported for such conditions [36].

In skeletal muscle, TH induce expression of *SLC2A4* encoding the glucose transporter GLUT4 as well as trafficking of GLUT4 to the plasma membrane [29]. Thus, increased glucose uptake in peripheral tissues, namely the muscle, might explain our observation that in spite of increased hepatic glucose release the corresponding serum concentrations were not significantly changed (Table 1). However, besides glucose, glycogenolysis also produces glucose-6-phosphate which subsequently can be converted to mannose [37]. Whether the increased plasma mannose levels observed in our study (Fig. 3) reflect TH-stimulated glycogenolysis in liver or result from glycogen breakdown in muscle remains unclear.

### A plasma metabolome signature indicating augmented defense against systemic oxidative stress

The strong positive FT_4_-association observed for γ-glutamyl amino acid (GGAA) levels represents one of the novel findings of this study (Fig. 3). As elevated GGAA levels were recently related to several types of liver damage [38] and TH were furthermore demonstrated to represent potent hepatic mitogens triggering hepatocyte turnover [39], we first hypothesized that the observed increase during thyrotoxicosis might also result from hepatocellular lysis. However, determination of the classical laboratory markers for liver damage, namely ALT, AST, and GGT activities in serum, did not support this hypothesis.

GGAA synthesis by transfer of the glutathione (GSH) glutamyl moiety to free amino acids is catalyzed by γ-glutamyl transpeptidase (GGT) as part of the γ-glutamyl cycle (GGC). As the GGT catalytic center is localized at the extracytoplasmic side of the hepatocyte membrane, this represents the site of γ-glutamyl amino acid production, which is strongly determined by the availability of GSH [40]. The rate-limiting step in GSH biosynthesis is catalyzed by the heterodimeric glutamate-cysteine ligase consisting of a heavy catalytic and a light regulatory subunit. Expression of *GCLC* and *GCLM* encoding the two subunits of the enzyme is induced by transcriptional up-regulation *via* NRF2, the redox-sensitive key regulatory transcription factor of the major cellular defense system against oxidative stress [41]. Strikingly, hepatic activation of the NRF2 regulon by TH-induced production of reactive oxygen species due to increased respiration was demonstrated in rodent models [42, 43]. Similarly, a stimulatory effect of TH on the GGC resulting in improved antioxidant capacity was shown in astrocytes [44]. Thus, it seems plausible that the increase in plasma GGAA levels under conditions of thyrotoxicosis observed in this study reflects the NRF2-mediated induction of GSH synthesis as part of the systemic network antagonizing pronounced oxidative stress as a consequence of FT_4_-stimulated respiration.

Our study further revealed an FT_4_-associated increase in n3 and n6 plasma PUFAs and a drop in LPs (Fig. 3), partially replicating findings from other recent metabolome analyses [17, 32, 45]. Indeed, the LPs displayed opposite associations, depending on the presence of either choline (lysophosphatidylcholine (LPC); positively associated) or ethanolamine (lysophosphatidylethanolamine (LPE); negatively associated) as the head group (Fig. 3).

Previous animal studies demonstrated a direct negative effect of TH on the hepatic PUFA content [46–48], wherein PUFAs were depleted under hyperthyroid conditions. It was supposed that TH initiate remodeling of mitochondrial membranes resulting in a decrease of PUFA-containing phosphatidylcholines. As saturated FAs are less prone to peroxidation, this was interpreted as an adaptive mechanism contributing to the protection of mitochondrial membranes against enhanced oxidative stress forced by TH-induced up-regulation of respiration [46, 47]. Thus, PUFA release from mitochondrial membranes might be reflected in the observed increased plasma PUFA levels.

The negative association between serum FT_4_ and plasma LPEs represents an additional novel finding of this study (Fig. 3). Previous analyses of rodent hyperthyroidism models [49, 50] revealed enhanced incorporation of phosphatidylethanolamine (PE) in mitochondria of liver [50] and brain [49]. Augmented PE utilization might explain the observed plasma decrease of PE metabolites, namely the LPEs.

### Discordant changes in classical and novel markers of kidney function under thyrotoxicosis

As outlined above, increased plasma creatine levels under conditions of thyrotoxicosis most likely reflect decreased creatine phosphorylation in skeletal muscle due to pronounced uncoupling of respiration and ATP synthesis. By contrast, we observed decreased plasma levels of the creatine catabolite creatinine in this study (Fig. 3), which could be explained by increased renal clearance of creatinine, possibly above the general glomerular filtration rate [51]. Both findings were previously described for hyperthyroidism [52, 53]. Thus, a reliable estimation of the glomerular filtration rate based on creatinine might be biased and hampers the interpretation of kidney function in thyroid disease. Similar holds true for CYTC, the second common circulating marker for kidney function, which was strongly elevated by L-T_4_ treatment in our study, a finding in line with previous results of hyperthyroidism studies [54, 55]. Very recently, novel promising markers for kidney function estimation were published using a similar metabolomics approach as in this study [56], namely C-mannosyltryptophan and pseudouridine. In contrast to creatinine and CYTC, none of these markers was altered to a similar extent in the present study (Additional file 2: Table S1), suggesting that the observed changes in creatinine and CYTC levels are metabolically driven ﻿and to a lesser extent due to altered kidney function. Thus, novel markers of kidney function such as C-mannosyltryptophan and pseudouridine may be advantageous under conditions of thyrotoxicosis.

### Thyrotoxicosis increases plasma asymmetric dimethylarginine (ADMA) levels

We observed increased plasma levels of methylated arginine (as the sum of ADMA and symmetric dimethylarginine) under L-T_4_ treatment, while the levels of its catabolite citrulline decreased (Fig. 3). A positive association between TH levels and circulating ADMA was previously reported in epidemiological studies and under conditions of hyperthyroidism [57–60]. ADMA was suggested as a severe risk factor for cardiovascular disease (for review see [61]), mainly as it directly inhibits endothelial nitric oxide synthase (NOS), thereby impairing NO-dependent vasodilation and favoring hypertension. ADMA is generated as a putative by-product of pronounced systemic proteolysis [62], which is known to be triggered by TH excess [63]. Therefore, the increased plasma levels of leucine, isoleucine, and their degradation intermediate 2-methylbutyrylcarnitine in the present study (Fig. 3) might indicate pronounced FT_4_-associated protein catabolism. The products of dimethylarginine dimethylaminohydrolase-catalyzed ADMA degradation, citrulline, and dimethylamine, are subsequently cleared by the kidneys [64]. At least one study using a murine model [65] described an inhibitory effect of prolonged T_3_ treatment on dimethylarginine dimethylaminohydrolase in the liver. Thus, our novel observation of an FT_4_-associated ADMA/symmetric dimethylarginine increase and citrulline decrease in plasma may indicate TH-induced suppression of ADMA catabolism leading to its systemic accumulation. In sum, augmented production as well as reduced decomposition of ADMA might therefore contribute to its increased plasma levels under conditions of thyrotoxicosis.

Thyrotoxicosis-induced elevated ADMA levels are predicted to mediate increased blood pressure by NOS inhibition [57–59]. The notion that TH directly affect vascular smooth muscle cells causing vascular relaxation and dilatation [66, 67] seemingly contradict the above discussed findings. However, it appears that the described TRα-dependent, non-genomic activation of endothelial NOS via the PI3/AKT-pathway is only present at very high TH concentrations [68, 69], which were not observed in the present study.

### General FT_4_-associated alterations of the plasma proteome

**Fig. 4:**
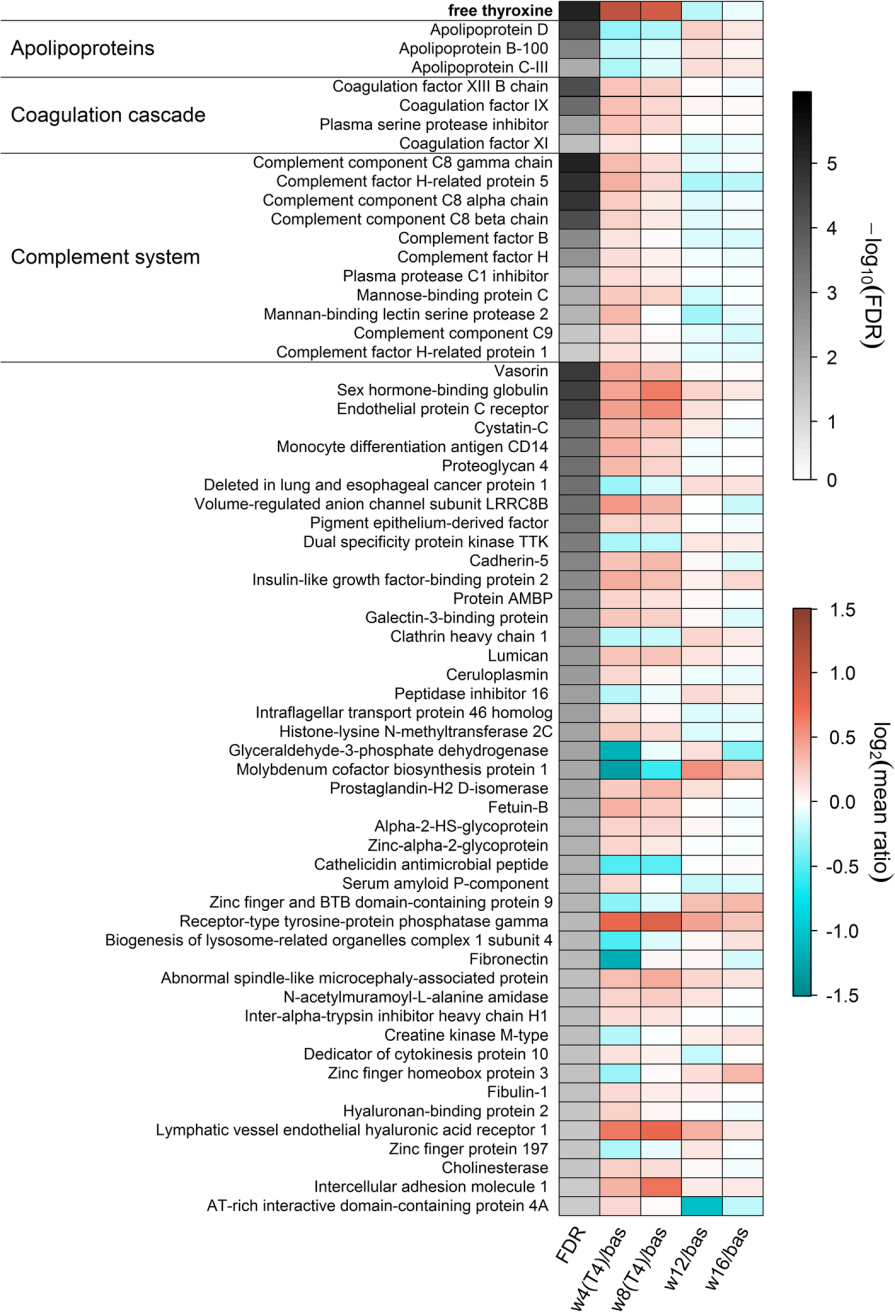
Heatmap of plasma proteins significantly associated with free thyroxine (FT_4_) in mixed effect linear regression models. The first column displays the values of the mean false discovery rate (FDR) for the FT_4_ effect across 101 subsamples of the data coded in grey color. The other columns indicate the mean log_2_-ratio from baseline (bas) compared to 4 (w4(T4)) and 8 (w8(T4)) weeks of treatment as well as 4 (w12) and 8 (w16) weeks after finishing the treatment. The time course of FT_4_ concentrations is shown on the top of the map as reference. Orange shading denotes an increase and blue shading a decrease compared to baseline, respectively. Derived physiological signatures are labeled on the left. The corresponding estimates and FDR values from regression analysis can be found in Additional file 2: Table S2

Using an untargeted shotgun-LC-MS/MS-approach, our proteome study demonstrated two major general categories of proteins exhibiting FT_4_-associated plasma levels, namely the higher abundant actively secreted proteins predominantly originating from the liver and representing the majority of detected proteins and, in addition, leakage proteins whose presence in the circulation is predicted to result from cell lysis. The latter comprised only 3% of the total protein intensity. Similar to the metabolome, the levels of 63 out of 437 detected proteins (14%) exhibited significant associations with FT_4_ (Additional file 2: Table S2). The majority (N = 47) was positively associated, whereas about one fourth (N = 16) demonstrated a negative association with serum FT_4_. SHBG and CYTC, which are known to be altered in thyroid dysfunction, were among the strong positively FT_4_-associated proteins. The changes in the levels of both proteins as determined by MS were similar to those measured with standard laboratory assays (Additional file 1: Figure S3). Of note, we observed no significant alterations of the major TH transport proteins thyroxine-binding globulin (SERPINA7) and thyroid-hormone binding protein transthyretin. The results are summarized in Fig. 4 and Additional file 2: Table S2.

### A plasma proteome signature indicating decreased lipoprotein particle levels during thyrotoxicosis

TH-dependent alterations in the levels of apolipoproteins and different lipid-rich particles were reported previously [70–73]. In line with these findings, we observed a significant drop in the plasma levels of apolipoproteins APOB (apoB-100), APOD, and APOC3 (apoCIII) during the peak of induced thyrotoxicosis, where APOD exhibited the strongest association (Fig. 4 and Additional file 2: Table S2).

The apoB100 protein represents the primary apolipoprotein of VLDL and LDL particles essentially mediating systemic transport of lipids including cholesterol to peripheral tissues in the context of the fuel and overflow transport pathways, respectively, and is the primary ligand of the low-density lipoprotein receptor (LDL-R) [74]. Peripheral as well as liver-specific LDL particle uptake via apoB100-dependent LDL-R binding and endocytosis is promoted by TH, as *LDLR* encoding this receptor represents a direct TR target and is additionally up-regulated by the transcriptional regulator SREBP-2, which, in turn, is also induced by TH at the gene expression level [75–77]. Thus, the decreased apoB100 abundance under conditions of thyrotoxicosis represents a direct consequence of TH-stimulated LDL uptake from the circulation.

APOD is primarily associated with HDL particles mediating reverse cholesterol transport (RCT) from peripheral tissues to the liver. It represents an atypical apolipoprotein and belongs to the family of lipocalin proteins which transport small hydrophobic ligands [78]. TH stimulate the RCT by increasing the expression of several genes involved in cholesterol metabolism, among them *SCARB1* encoding the multiple-ligand binding scavenger receptor class B member 1 (SRB1), which is responsible for the binding of cholesterol enriched HDL particles in numerous cell tissues, namely liver and adrenal [79]. Therefore, the observed drop in APOD plasma levels can be explained by TH-stimulated HDL particle binding as part of the activated RCT.

The apoCIII protein is localized on the surface of mature triglyceride-rich chylomicrons and VLDL particles as well as HDL particles contributing to the fuel transport and the RCT pathways, respectively [74]. Uptake of VLDL particles is mediated by the VLDL receptor that binds APOE, a further apolipoprotein found in chylomicrons. Expression of the gene encoding this receptor was demonstrated to be under positive TH control in a rodent model [80]. Therefore, the decreased apoCIII levels observed in our study during thyrotoxicosis are explained by TH-mediated up-regulation of the genes encoding VLDL receptor and SRB1.

The TH-induced drop in the plasma levels of apolipoproteins belonging to different classes is consistent with the observed significant TH-associated transient reductions in the plasma levels of HDL-cholesterol, LDL-cholesterol, and total cholesterol as determined by standard clinical assays (Table 1 and Fig. 2).

### A plasma proteome signature indicating augmented coagulation during thyrotoxicosis

The positive association between blood coagulation and TH concentrations is well known. In line with the predominantly clinical studies published so far on this topic [81–85], our proteome analysis demonstrated several proteins involved in the coagulation cascade to exhibit FT_4_-associated plasma levels (Fig. 4); these findings have been published separately [86]. In short, we were able to demonstrate that experimental thyrotoxicosis increases the levels of coagulation cascade proteins in plasma, supporting a positive impact of TH on blood coagulation even at non-pathological levels. Coagulation factor XIII B chain (F13B) and the factors IX (FA9) and XI (FA11) exhibited significantly increased levels under thyrotoxicosis conditions, as well as SERPINA5 (IPSP), an inhibitor of activated protein C. As the latter inhibits clot formation, increased levels of its inhibitor SERPINA5 and of factors XIIIB, IX, and XI can be predicted to result in a prothrombotic, hypercoaguable environment, in accordance with the aforementioned findings from clinical studies [81–85]. For factors XIIIB and IX, the increased plasma levels after L-T_4_-treatment were independently validated by ELISA techniques [86]. The molecular mechanism(s) underlying the increased levels of these proteins are currently not clear and cannot be clarified by our study design. Plausible hypotheses might be a TH-stimulated increase of their stability in plasma or enhanced expression of their encoding genes. The recently published finding of unaltered coagulation parameters in RTHβ patients despite elevated FT_4_ levels favors the latter hypothesis and suggests that the procoagulant effects observed under conditions of thyrotoxicosis and hyperthyroidism are mediated via TRβ [87].

### A plasma proteome signature indicating increased complement system plasma protein levels

Nine proteins of the complement system were positively associated with FT_4_, including mannose-binding protein C (MBL2) and mannan-binding lectin serine protease 2 (MASP2). Additionally, the complement-factor H-related proteins CFHR1 and CFHR5 as binding proteins of complement component C3b showed a positive association with serum FT_4_ (Fig. 4). Consistently, although the C3 protein barely missed statistical significance in the proteome analysis (Additional file 2: Table S2), higher abundance of C3 as well as C4 was determined by standard laboratory assays (Table 1 and Fig. 2). In addition, our metabolome analysis detected one C3 fragment (HWESASLLR) [88] mirroring the positive association (Fig. 3). Similarly, clinical studies [84, 89, 90] reported positive associations between complement system proteins and TH levels. Of note, the duration of hyperthyroidism might be crucial in this context, since a short-term model of experimental thyrotoxicosis revealed no significant C3 level alterations [84]. Data explaining the impact of TH on the complement system proteins, which originate primarily from the liver, are scarce. Previous studies demonstrated that *MBL2* encoding mannose-binding lectin, an early module of the lectin pathway in the complement system that exhibited increased plasma levels under thyrotoxicosis conditions in our study, represents a direct PPARα target [91, 92]. As PPARα belongs to those transcriptional co-regulators interacting with TH-ligated TR [29], it may be speculated that a corresponding TH-mediated induction of *MBL2* expression causes the observed plasma level increase, which might also hold true for other complement system proteins. However, in mice, the fact that several analyses of the hepatic transcriptome in different murine models before and after T_3_ application failed so far to demonstrate convincingly differentially expressed genes encoding complement system proteins [65, 93–95] argues against a TR-dependent regulation at the mRNA level. Future analyses involving TH-treated primary human hepatocytes might represent a promising approach to clarify the molecular mechanism(s) underlying the observed plasma level alterations of complement system proteins.

### Differences in response profiles and effect magnitudes

Different kinetic patterns as well as mean magnitude sizes were observed for several associated molecule categories (Figs. 3 and 4): ACs exhibited an invariable response during the complete study period whereas the levels of long-chain FAs further increased between 4 and 8 weeks (Fig. 3). In contrast, complement system proteins reached their maximal mean levels after 4 weeks of L-T_4_ treatment followed by a moderate decline after 8 weeks (Fig. 4). Time-dependent alterations were also evident in the magnitudes of the detected effects. For instance, lipid species demonstrated an almost two-fold increase, whereas the levels of complement system proteins increased only moderately (~30%). Despite these differences, all associations were most likely mediated by L-T_4_ treatment as they were captured by the mixed-effect linear regression models.

### A biomarker-based signature to predict thyroid function

**Fig. 5:**
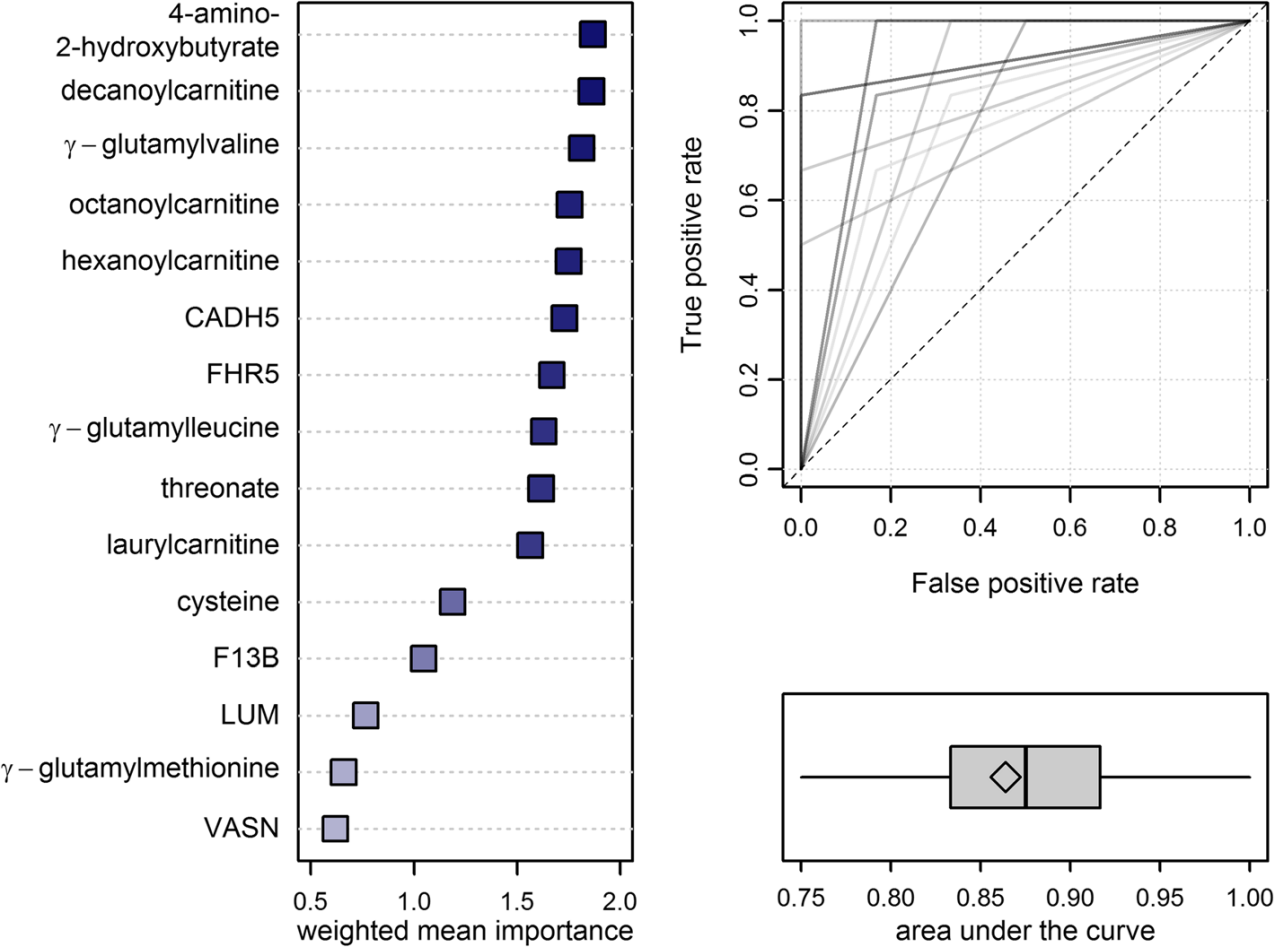
Final results from classification analyses using random forests in a two-stage cross-validation scheme with 50 inner and 30 outer loops (Additional file 1: Figure S1). *Left panel*: Fifteen most important metabolites/proteins ranked by a weighted (area under the curve) mean Gini index. *Right panel*: Receiver operating characteristic (ROC) curves (upper) and boxplot of the area under the curve (lower) from 30 outer loops. Overlapping ROCs are displayed by darker shades and the diamond indicates the mean AUC. *CADH5* cadherin-5, *FHR5* complement factor H-related protein 5, *F13B* coagulation factor XIII B chain, *LUM* lumican, *VASN* vasorin

Our approach of a precisely controlled transient increase in systemic TH levels in healthy male volunteers allowed characterizing effects clearly and unambiguously based on a defined duration and dose of thyroxine. As we deliberately searched for a clinically relevant and yet unmet diagnostic approach to classify TH status without using TSH and/or free TH, we combined the data generated by the untargeted OMICS techniques in a classification model. For this purpose, we built a random forest classifier via a two-stage cross-validation procedure (see Methods and Additional file 1: Figure S1), thereby allowing for a more realistic estimation of the generalization of the random forest than conventional k-fold cross-validation. This is exemplarily demonstrated by the ROC curves (Fig. 5), which were obtained when both situations occurred, perfect (AUC = 1) as well as fair (AUC = 0.75) classification neither representing the expected general performance. Additionally, the approach chosen here allowed for combining classification with feature selection to define a small set of biomarkers among the numerous FT_4_-associated molecules described above. We obtained a list comprising 15 metabolites and proteins (Fig. 5, left panel) exhibiting robust and good classification performance over all 30 validation runs (mean AUC = 0.86; Fig. 5, right panel) and therefore representing appropriate biomarkers for a reliable prediction of the TH status. Of this biomarker panel, most components were already discussed above in the context of different physiological signatures, namely increased resting energy expenditure and enhanced mitochondrial fatty acid β-oxidation (decanoylcarnitine, octanoylcarnitine, hexanoylcarnitine, laurylcarnitine), increased GSH synthesis as part of the augmented defense against oxidative stress as a consequence of stimulated respiration (γ-glutamylvaline, γ-glutamylleucine, γ-glutamylmethionine), and increased levels of complement system (FHR5) or coagulation (F13B) proteins.

Cadherin-5 (CDH5), also named VE-cadherin, represents a calcium-dependent adhesion protein specific to endothelial cells and is a major component of adherens junctions found in blood vessels. It is essentially involved in the regulation of vascular integrity, restricting permeability of the endothelium [96]. Shedding of CDH5 by proteinases increases microvascular permeability and is one of the molecular mechanisms involved in transendothelial neutrophil migration during inflammatory processes and endothelial apoptosis [97]. CDH5 shedding is mediated by two proteinases of the ADAM (A Disintegrin And a Metalloprotease domain metalloproteases) family, namely ADAM9 and ADAM10 [98], where the proteolytic activity of ADAM10 is stimulated by thrombin (coagulation factor 2, F2a) [99]. Therefore, the observed increased levels of circulating CDH5 most probably result from stimulated thrombin activation under the prothrombotic thyrotoxicosis-associated conditions described above.

A similar explanation can be found for the increased vasorin (VASN) levels – VASN is a typical type I membrane protein that was identified as target of the ADAM17 metalloprotease. Limited proteolysis by ADAM17 generates a soluble fragment encompassing the extracellular VASN domain, which directly binds to transforming growth factor (TGF)β and attenuates TGFβ signaling [100]. As in the case of ADAM10, ADAM17-dependent shedding has been demonstrated to be stimulated by thrombin [101]. Consistently, the three CDH5-specific peptides as well as the six VASN-specific peptides that were detected in the proteome analysis exclusively mapped to the extracellular domains of the two proteins. Therefore, together with CFHR5, CDH5, and VASN can be assigned to the signature of increased levels of coagulation factors.

The plasma level of cysteine was previously reported to be decreased by TH-treatment via direct down-regulation of the expression of *CTH* and *CBS* encoding cystathionine gamma-lyase and cystathionine beta-synthase, respectively [102]. This might explain the pronounced negative association between the plasma levels of cysteine as well as its metabolite 4-amino-2-hydroxybutyrate and FT_4_ in our study (Fig. 3). Threonate, which is either derived from glycated proteins or represents a degradation product of ascorbate [103], was previously linked to altered thyroid function in two murine models [45, 104], but not yet in humans.

Finally, lumican (LUM) represents an extracellular matrix protein modified as a proteoglycan in several tissues. The core protein with leucine-rich repeats that are characteristic for the corresponding superfamily binds collagen fibrils and regulates its structure. In addition, LUM associates with CD14 on the surface of macrophages and neutrophils and promotes the CD14-TLR4 mediated response to bacterial lipopolysaccharides where it is involved in macrophage-mediated phagocytosis [105–107]. LUM is proteolytically degraded by matrix metalloproteinase (MMP) 14 [108], whose encoding gene *MMP14*, among several other genes encoding MMPs, was demonstrated to be down-regulated by TH [109]. Thus, the observed increase in plasma LUM levels might be the consequence of its decreased degradation due to TH-mediated reduced *MMP14* expression.

## Conclusion

The unique feature of the present study is its specific experimental design. By focusing on healthy young male volunteers we excluded any potentially interfering, disease-specific influences related, for example, to the autoimmune process in Graves’ thyrotoxicosis. This approach allows to control the extent and duration of biochemical hyperthyroidism induced by thyroxine as one of the most prescribed drugs and to monitor its recovery, thereby enabling unique insights into the kinetics of subjective changes experienced by the volunteers in relation to the biochemical changes measured.

Application of L-T_4_ clearly induced biochemical thyrotoxicosis in our volunteer sample as indicated by strongly suppressed TSH and clearly increased TH levels, which were rapidly reversed after ceasing hormone application. The model was further validated by known thyrotoxic alterations in biochemical markers, including SHBG, CYTC, and total and LDL-cholesterol [12, 13, 55].

Interestingly, as described in a previous study analyzing the same volunteer sample, subjectively the volunteers did not notice any thyrotoxic symptoms, which was supported by the negative results of a standardized questionnaire for thyrotoxicosis and a battery of behavioral and cognitive tests [24]. A 24-h blood pressure profile before and at the end of 8 weeks of thyroxine application was not significantly different, neither when analyzed over 24 h or during sleep between 01:00 and 06:00 am [24]. In contrast, comparison of the mean 24-h pulse rates revealed a moderate increase of about 8.9 beats/minute (from 66.7 to 75.6 beats/minute), which was not recognized by the volunteers [24].

L-T_4_ application clearly changed a large number of biochemical parameters beyond the expected alterations in TSH and TH levels or in known biochemical markers. The combined findings reveal a surprising discrepancy between biochemical alterations and subjective symptoms in this cohort of young healthy subjects and shed new light on the mechanisms mediating adaptation to subclinical hyperthyroidism. They suggest that biochemical alterations might be detectable considerably earlier than clinical symptoms occur and are much more sensitive. In consequence, the diagnosis of thyrotoxicosis in clinical routine might frequently miss the real onset time of the disease by several months.

The comprehensive robust analysis of a human thyrotoxicosis model using state of the art untargeted plasma OMICS approaches demonstrated a strong and pleiotropic metabolic impact of TH on this compartment. The characterized physiological signatures comprised biomarkers indicating increased resting energy expenditure, augmented defense against systemic oxidative stress, decreased lipoprotein particle levels, and increased levels of complement system proteins as well as coagulation factors, where the latter results in a pro-thrombotic environment. Measurement of 15 specific biomarkers, metabolites as well as proteins, allowed reliable prediction of the individual thyroid function in the analyzed sample independent of common TSH and FT_4_ measurements. In addition, by following the subjects during the recovery from thyrotoxicosis we gained first insight in the target-specific kinetics of TH-dependent responses. The definition of this prediction panel might represent an important step forward in molecular characterization of early forms of hyperthyroidism. However, it has to be emphasized that the analyzed study sample consisted exclusively of young men, limiting the generalizability of the results. Therefore, further validation studies using larger samples of higher complexity in terms of age and sex as well as hypothyroid conditions have to be performed. Similarly, our findings concerning the influence of increased TH levels on kidney function markers and systemic ADMA levels that might be of special clinical importance have to be replicated in appropriate patient cohorts and/or population-based studies where the required parameters are available.

## Additional files

Additional file 1: Figure S1. Flowchart of the classification procedure. Each outer loop started with splitting off the validation data from the remaining data that were further divided in a training set and a test set at each start of a training period. The training set was used to build a random forest (RF) exploiting all metabolites/proteins as features. Predictions were made on the test set and RF performance was assessed by the area under the curve (AUC) while feature importance was measured as the Gini index. Training was repeated on i different splits of data and an AUC-weighted mean Gini index was computed for all features. Afterwards, a new RF restricted to the top k features (those with the highest mean Gini index) was built. It was trained on the combination of training and test data and employed to classify the validation data. The described procedure was repeated j-times, once more yielding an AUC-weighted mean Gini index for feature importance. The final results of this procedure with i = 50, j = 30 and k = 15 are shown in Fig. 4 in the main text. **Figure S2.** Boxplots for each study time point for glucose and total cholesterol levels measured either by standard laboratory assays (dark grey) or by metabolomics (light grey). *bas* baseline, *w4(T4)/w8(T4)* 4 and 8 weeks of levothyroxine treatment; *w12/w16* 4 and 8 weeks after stopping the application. **Figure S3.** Boxplots for each study time point for sexhormone- binding globulin (SHBG) and cystatin C levels determined either by standard laboratory assays (dark grey) or in the untargeted proteome approach (light grey). *bas* baseline, *w4(T4)/w8(T4)* 4 and 8 weeks of levothyroxine treatment, *w12/w16* 4 and 8 weeks after stopping the application. (DOCX 580 kb)
Additional file 2: Table S1. Results from mixed-effect linear regression analyses with serum FT4 concentrations as exposure and metabolites as outcome. **Table S2.** Results from mixed-effect linear regression analyses with serum FT4 concentrations as exposure and protein levels as outcome. (XLSX 88 kb)

## Abbreviations

AC: acyl carnitines
ADMA: asymetric dimethylarginine
ALT: alanine amino transferase
APOB: apolipoprotein B100
APOC3: apolipoprotein C3
APOD: apolipoprotein D
AST: aspartate amnio transferase
AUC: area under the curve
CDH5: cadherin-5
CPT-I: carnitine palmitoyltransferase-I
CYTC: cystatin C
FDR: false discovery rate
FFA: free fatty acids
FT_3_: (free) triiodothyronine
FT_4_: (free) thyroxine
GC: gas chromatography
GGAA: γ-glutamyl amino acid
GGC: γ-glutamyl cycle
GGT: γ-glutamyl transpeptidase
GSH: glutathione
HDL: high-density lipoprotein
LC: liquid chromatography
LDL: low-density lipoprotein
LP: lysolipids
LPC: lysophosphatidylcholine
LPE: lysophosphatidylethanolamine
L-T_4_: levothyroxine
LUM: lumican
MS: mass spectrometry
NOS: nitric oxide synthase
PE: phosphatidylethanolamine
PUFA: poly unsaturated fatty acids
RCT: reverse cholesterol transport
ROC: receiver operating characteristics
RTHβ: resistance to thyroid hormone
SHBG: sex hormone-binding globulin
TH: thyroid hormones
TR: thyroid hormone receptor
TRH: thyrotropin releasing hormone
TSH: thyrotropin
VASN: vasorin
VLDL: very low-density lipoprotein
