# Supplementary material for: Plasma proteome and metabolome characterization of an experimental human thyrotoxicosis model
Source: BMC Med. 2017 Jan 9;15:6. doi: 10.1186/s12916-016-0770-8 (PMC5220622; doi:10.1186/s12916-016-0770-8)
Supplement: Additional file 1: Figure S1. — Flowchart of the classification procedure. Each outer loop started with splitting off the validation data from the remaining data that were further divided in a training set and a test set at each start of a training period. The training set was used to build a random forest (RF) exploiting all metabolites/proteins as features. Predictions were made on the test set and RF performance was assessed by the area under the curve (AUC) while feature importance was measured as the Gini index. Training was repeated on i different splits of data and an AUC-weighted mean Gini index was computed for all features. Afterwards, a new RF restricted to the top k features (those with the highest mean Gini index) was built. It was trained on the combination of training and test data and employed to classify the validation data. The described procedure was repeated j-times, once more yielding an AUC-weighted mean Gini index for feature importance. The final results of this procedure with i = 50, j = 30 and k = 15 are shown in Fig. 4 in the main text. Figure S2. Boxplots for each study time point for glucose and total cholesterol levels measured either by standard laboratory assays (dark grey) or by metabolomics (light grey). bas baseline, w4(T4)/w8(T4) 4 and 8 weeks of levothyroxine treatment; w12/w16 4 and 8 weeks after stopping the application. Figure S3. Boxplots for each study time point for sexhormone- binding globulin (SHBG) and cystatin C levels determined either by standard laboratory assays (dark grey) or in the untargeted proteome approach (light grey). bas baseline, w4(T4)/w8(T4) 4 and 8 weeks of levothyroxine treatment, w12/w16 4 and 8 weeks after stopping the application. (DOCX 580 kb) [file 12916_2016_770_MOESM1_ESM.docx]

**Supplement**

**
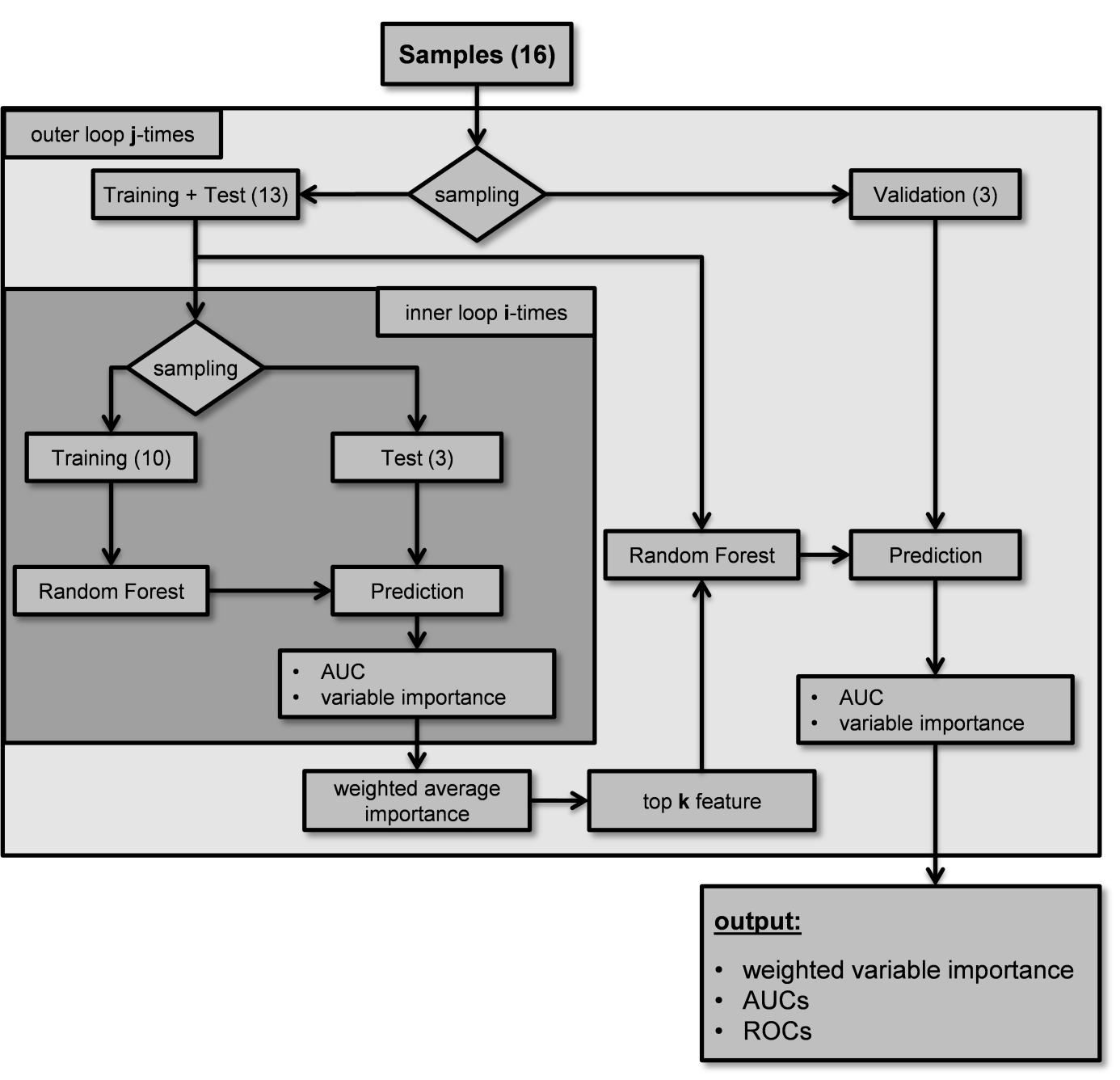
**

**Figure S1.** Flowchart of the classification procedure. Each outer loop started with splitting off the validation data from the remaining data that were further divided in a training set and a test set at each start of a training period. The training set was used to build a random forest (RF) exploiting all metabolites/proteins as features. Predictions were made on the test set and RF performance was assessed by the area under the curve (AUC) while feature importance was measured as the Gini index. Training was repeated on **i** different splits of data and an AUC-weighted mean Gini index was computed for all features. Afterwards, a new RF restricted to the top *k* features (those with the highest mean Gini index) was build. It was trained on the combination of training and test data and employed to classify the validation data. The described procedure was repeated **j**-times, once more yielding an AUC-weighted mean Gini index for feature importance. The final results of this procedure with i=50, j=30 and k=15 are shown in Figure 4 in the main text.

**
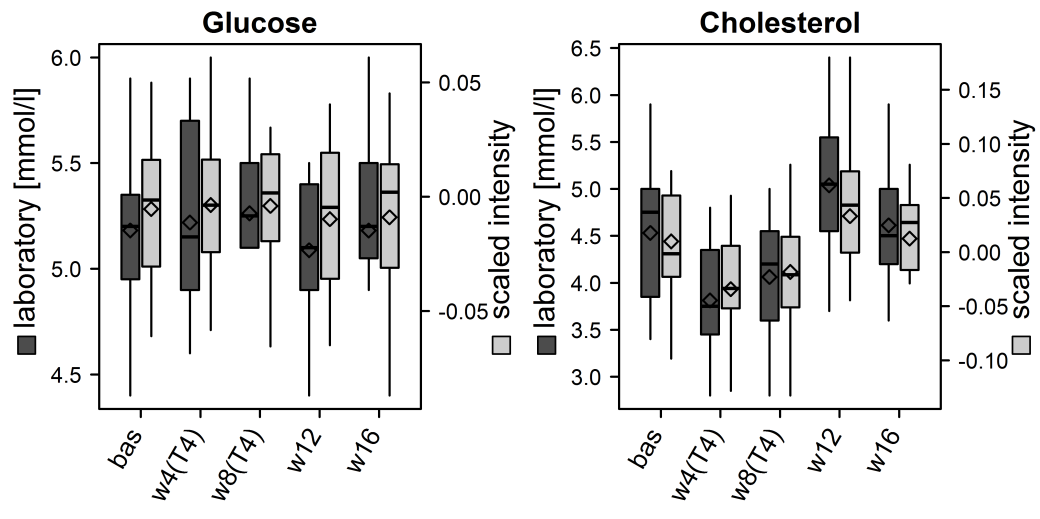
**

**Figure S2** Boxplots for each study time point for glucose and total cholesterol levels measured either by standard laboratory assays (dark grey; Dimension VISTA, Siemens Healthcare Diagnostics, Eschborn, Germany) or by metabolomics (light grey; Metabolon Inc., Durham, NC, USA). bas = baseline; w4(T4)/w8(T4) = four and eight weeks of levothyroxine treatment; w12/w16 = four and eight weeks after stopping the application


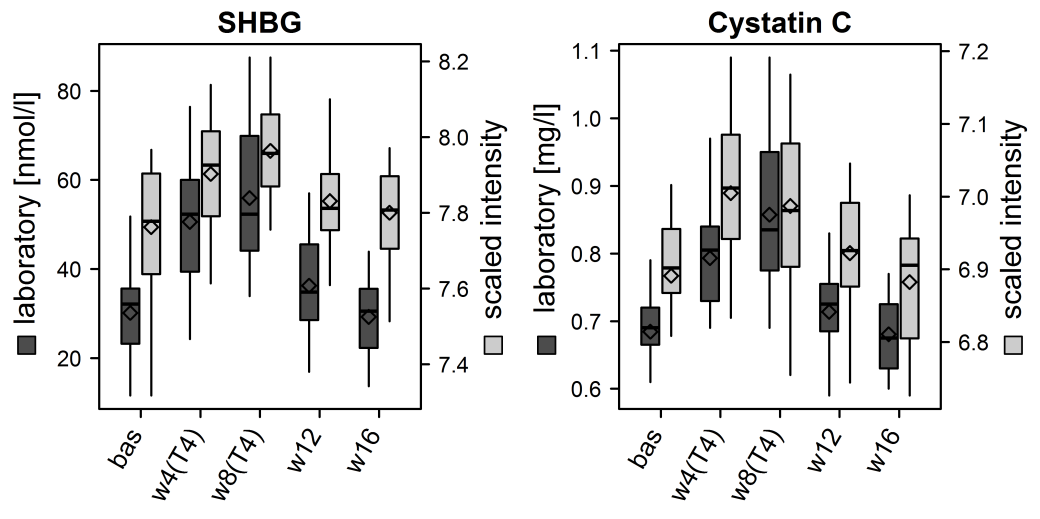


**Figure S3** Boxplots for each study time point for sex-hormone binding globulin (SHBG) and cystatin C levels determined either by standard laboratory assays (dark grey; SHBG: Immulite 2000, Siemens Healthcare Medical Diagnostics, Bad Nauheim, Germany; cystatin C: Dimension VISTA, Siemens Healthcare Diagnostics, Eschborn, Germany) or in the untargeted proteome approach (light grey). bas = baseline; w4(T4)/w8(T4) = four and eight weeks of levothyroxine treatment; w12/w16 = four and eight weeks after stopping the application
